# Cardiovascular safety of Janus kinase inhibitors: A pharmacovigilance study from 2012–2023

**DOI:** 10.1371/journal.pone.0322849

**Published:** 2025-05-12

**Authors:** Xiaoyan Zhong, Jianchun Luo, Yuexi Huang, Shurong Wang, Yilan Huang

**Affiliations:** 1 Department of Pharmacy, The Affiliated Hospital, Southwest Medical University, Luzhou, China; 2 Department of Critical Care Medicine, The Affiliated Hospital, Southwest Medical University, Luzhou, China; PearResearch / Government Doon Medical College, INDIA

## Abstract

Janus kinase inhibitors (JAKinibs) are increasingly used for autoimmune diseases, prompting concerns about their cardiovascular safety. This study aims to assess the cardiovascular safety of JAKinibs in real-world settings. We conducted a retrospective analysis of FDA Adverse Event Reporting System (FAERS) data from the fourth quarter of 2012 to the second quarter of 2023, focusing on cardiovascular adverse events (AEs) associated with JAKinibs. We used disproportionality analysis to calculate reporting odds ratios (RORs) and identify signals of increased cardiovascular risk. This study identified 13,556 reports of cardiovascular AEs associated with JAKinibs in the FAERS database. Compared to the full database, Baricitinib exhibited significant signals for embolic and thrombotic events (ROR_025_ = 5.58), ischemic heart disease (ROR_025_ = 1.56), and cardiac arrhythmias (ROR_025_ = 1.14). Tofacitinib was associated with the signal for hypertension (ROR_025_ = 1.05), and upadacitinib was linked to embolic and thrombotic events (ROR_025_ = 1.23). When compared to TNF-α inhibitors, upadacitinib, baricitinib and tofacitinib showed 7, 6, and 2 positive signals, respectively (all ROR_025_ > 1). These findings highlight the need for careful cardiovascular monitoring and risk assessment for patients receiving JAKinibs, particularly those with pre-existing cardiovascular risk factors or older age.

## 1. Introduction

JAK inhibitors (JAKinibs), which target the JAK-STAT pathway, have revolutionized the treatment of autoimmune and inflammatory diseases. These agents have shown substantial efficacy in reducing disease activity and improving the quality of life for patients with conditions like RA, psoriasis, and inflammatory bowel disease [1,2]. FDA-approved drugs like tofacitinib, baricitinib, and upadacitinib have shown significant clinical benefits [3–5]. However, safety concerns, particularly cardiovascular risks, have raised questions about their long-term use. While early studies did not show an increased risk of major adverse cardiovascular events (MACE) [6], the ORAL Surveillance trial found higher cardiovascular risks with tofacitinib compared to Tumor necrosis factor alpha (TNF-α) inhibitors [7]. This led to FDA-mandated boxed warnings for JAKinibs in 2021 [8]. This highlights the importance of post-marketing surveillance to detect risks that may not be seen in clinical trials.

The FDA Adverse Event Reporting System (FAERS), with over 28 million reports, is a powerful tool for identifying rare or delayed drug side effects [9]. Despite issues like underreporting, its large scale helps spot safety signals that can inform clinical practice [10]. For example, it has linked biologics to stroke and GLP-1 agonists to suicidal behaviors [11,12].

This study uses FAERS data to map cardiovascular side effects associated with JAKinibs. By creating a signal spectrum plot, we aim to clarify risk patterns, support evidence-based clinical decisions, and improve post-marketing surveillance strategies for this important class of drugs.

## 2. Materials and methods

### 2.1 Data sources and procedures

This study analyzed cardiovascular events linked to JAKinibs using data from Q4 2012 to Q2 2023. To manage duplicate entries in FAERS, we applied a deduplication process. Specifically, for matching case IDs, we retained the record with the most recent FDA_DT date. If both case ID and FDA_DT matched, we selected the entry with the highest primary ID.

We expanded JAKinibs-related adverse events (AEs) data by cross-referencing generic/trade names and chemical identifiers from the Drugs@FDA database. All AEs were coded using MedDRA Preferred Terms (PTs), which map to higher-level categories (HLT, HLGT, SOC). Cardiovascular events were identified through eight predefined narrow Standardized MedDRA Queries (SMQs): cardiac arrhythmias, cardiac failure, cardiomyopathy, embolic/thrombotic events, hypertension, ischemic heart disease, pulmonary hypertension, and QT prolongation/torsade de pointes. These SMQs align with established cardiovascular toxicity assessment criteria from prior research (Table 1).

**Table 1:** Cardiovascular adverse events grouped into 8 narrow categories of SMQs according to MedDRA 24.0.

SMQ Name	SMQ Code
Embolic and thrombotic events	20000081
Ischaemic heart disease	20000043
Pulmonary hypertension	20000130
Torsade de pointes/ QT prolongation	20000001
Hypertension	20000147
Cardiac failure	20000004
Cardiac arrhythmias	20000049
Cardiomyopathy	20000150

Given the FDA’s boxed warning revisions for tofacitinib, baricitinib, and upadacitinib, this study focused on these three JAKinibs to investigate cardiovascular events. We extracted relevant reports and collected data on caseid, primaryid, demographics, reporting country, dates (START_DT, EVENT_DT, FDA_DT), outcomes, concomitant medications, indications, PT, and other pertinent details. Patients using lipid-lowering drugs, antihypertensives, antihyperglycemics, or anticoagulants were classified as having pre-existing cardiovascular disease. The onset time of AEs was calculated from START_DT to EVENT_DT, and AEs resulting in death, life-threatening situations, hospitalization, prolonged hospitalization, disability, or other significant medical events were defined as serious AEs.

### 2.2 Data analysis

Disproportionality analysis, a common signal detection method in pharmacovigilance, helps identify potential associations between AEs and specific drugs. In this study, we used the Reporting Odds Ratio (ROR) method, known for its simplicity and sensitivity, to evaluate the potential association between cardiovascular adverse reactions and JAK inhibitors. RORs were calculated using a 2x2 contingency table to compare the proportion of cardiovascular AEs associated with JAK inhibitors to those in the entire FAERS database and to reports involving TNF-α inhibitors (S1 Table). A signal was defined as an ROR025 value greater than 1, accompanied by at least three reports of the specific AE. This criterion ensures that the association between the drug and the AE is statistically significant and not due to random reporting. The onset time of cardiovascular AEs was assessed through Kruskal-Wallis test analysis. All data processing was performed using R version 3.2. The calculation formula for the ROR and 95% confidence interval (CI) is as follows:


$$ROR=\frac{ac}{bd}$$

95

## 3. Results

### 3.1 Descriptive analysis

**Fig 1:**
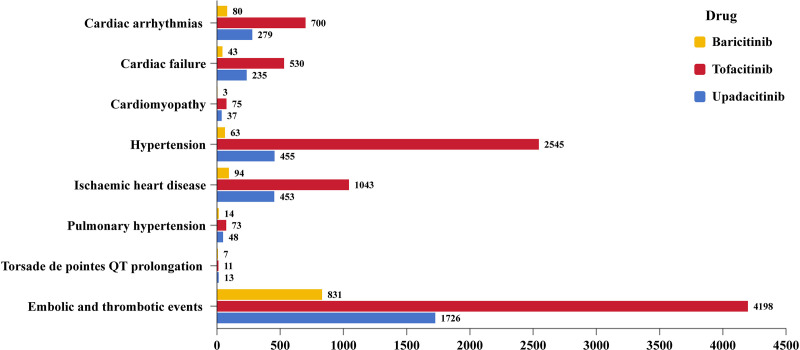
The bar plot shows the number of reports with cardiovascular AEs for different JAKinibs treatment.

**Fig 2:**
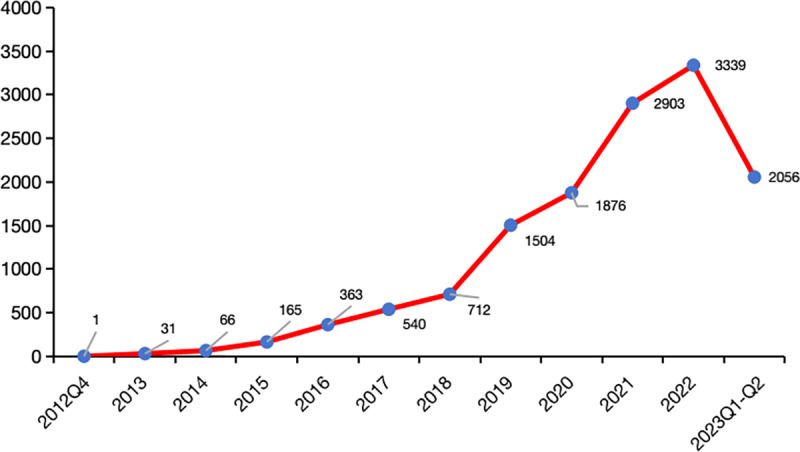
The time trend of cardiovascular AEs.

The database contains a total of 13,556 reports on cardiovascular adverse reactions associated with JAKinibs, representing 3.1% of all AEs linked to these inhibitors. The types of cardiovascular AEs varies across different types of JAKinibs. Specifically, there were 9,175 reports implicating tofacitinib, 3,246 reports for upadacitinib, and 1,135 reports for baricitinib. The distribution of cardiovascular AEs for different JAKinibs treatment is illustrated in Fig 1. From the fourth quarter of 2012 to the second quarter of 2023, there was a consistent annual increase in the number of reports over the years(Fig 2).

In these cardiovascular AEs linked to JAKinibs, 71.92% of patients were aged 50 years or older, with 39.19% being over 65 years old. The female patients outnumbered the male patients at 67.61%, and this gender difference was statistically significant. Those with a pre-existing cardiovascular disease history accounted for 20.53% of cases, while patients with a history of diabetes were 3.48% of the total reported cases. Among the reported cardiovascular AEs, hospitalization and other serious events were most common, with 874 reports of death, 187 reports of disability, and 513 reports classified as life-threatening (Table 2).

**Table 2:** The clinical characteristics of cardiovascular AEs.

	Total	Hypertension	Cardiac arrhythmias	Cardiac failure	Cardiomyopathy	Embolic and thrombotic events	Ischaemic heart disease	Pulmonary hypertension	Torsade de pointes/QT prolongation
**Number of AE reports**	13556	3063	1059	808	115	6755	1590	135	31
**Ages**	51.41(26.97)	52.67(23.71)	53.06(27.73)	54.59(28.47)	50.95(27.02)	48.94(28.11)	52.71(26.38)	50.16(26.18)	51.13(26.69)
<18 years	9	3	0	0	0	5	0	1	0
18 to 64 years	5690	1638	368	248	56	2656	647	63	14
≥65 years	5312	998	492	407	37	2660	658	48	12
Missing	2545	424	199	153	22	1434	285	23	5
**Sex**									
Female	9165(67.61)	2448(79.92)	744(70.25)	545(0.67)	71(0.62)	4304(0.64)	943(0.59)	95(0.7)	15(0.48)
Male	3797(28.01)	572(18.67)	284(26.82)	231(0.29)	38(0.33)	2046(0.3)	576(0.36)	35(0.26)	15(0.48)
Missing	594(4.38)	43(1.4)	31(2.93)	32(0.04)	6(0.05)	405(0.06)	71(0.04)	5(0.04)	1(0.03)
**Primary Suspect Drug**									
tofacitinib	9175(67.68)	2545(83.09)	700(66.1)	530(0.66)	75(0.65)	4198(0.62)	1043(0.66)	73(0.54)	11(0.35)
upadacitinib	3246(23.95)	455(14.85)	279(26.35)	235(0.29)	37(0.32)	1726(0.26)	453(0.28)	48(0.36)	13(0.42)
baricitinib	1135(8.37)	63(2.06)	80(7.55)	43(0.05)	3(0.03)	831(0.12)	94(0.06)	14(0.1)	7(0.23)
**Indications**									
Rheumatoid arthritis	8126(59.94)	1850(60.4)	676(63.83)	540(0.67)	74(0.64)	3849(0.57)	1015(0.64)	101(0.75)	21(0.68)
Psoriatic arthropathy	432(3.19)	134(4.37)	45(4.25)	20(0.02)	0(0)	177(0.03)	49(0.03)	6(0.04)	1(0.03)
Colitis ulcerative	485(3.58)	98(3.2)	31(2.93)	15(0.02)	4(0.03)	291(0.04)	45(0.03)	1(0.01)	0(0)
COVID-19	436(3.22)	4(0.13)	39(3.68)	15(0.02)	1(0.01)	351(0.05)	16(0.01)	7(0.05)	3(0.1)
other	1235(9.11)	193(6.3)	79(7.46)	65(0.08)	13(0.11)	721(0.11)	154(0.1)	8(0.06)	2(0.06)
Missing	2842(20.96)	784(25.6)	189(17.85)	153(0.19)	23(0.2)	1366(0.2)	311(0.2)	12(0.09)	4(0.13)
**Reporting countries**									
US	8999(66.38)	2461(80.35)	702(66.29)	488(0.6)	73(0.63)	4296(0.64)	889(0.56)	73(0.54)	17(0.55)
Missing	768(5.67)	85(2.78)	83(7.84)	59(0.07)	6(0.05)	394(0.06)	121(0.08)	14(0.1)	6(0.19)
DE	362(2.67)	28(0.91)	45(4.25)	30(0.04)	9(0.08)	180(0.03)	53(0.03)	14(0.1)	3(0.1)
JP	251(1.85)	12(0.39)	9(0.85)	40(0.05)	2(0.02)	142(0.02)	43(0.03)	3(0.02)	0(0)
CA	1174(8.66)	264(8.62)	95(8.97)	82(0.1)	9(0.08)	527(0.08)	188(0.12)	8(0.06)	1(0.03)
BE	38(0.28)	2(0.07)	1(0.09)	1(0)	1(0.01)	29(0)	1(0)	2(0.01)	1(0.03)
FR	175(1.29)	9(0.29)	8(0.76)	8(0.01)	2(0.02)	119(0.02)	25(0.02)	3(0.02)	1(0.03)
GB	160(1.18)	10(0.33)	5(0.47)	7(0.01)	0(0)	112(0.02)	24(0.02)	2(0.01)	0(0)
other	1629(12.02)	192(6.27)	111(10.48)	93(0.12)	13(0.11)	956(0.14)	246(0.15)	16(0.12)	2(0.06)
**Reporter**									
Consumer	6474(47.76)	1509(49.27)	521(49.2)	412(0.51)	41(0.36)	3098(0.46)	829(0.52)	55(0.41)	9(0.29)
Physician	2858(21.08)	383(12.5)	188(17.75)	168(0.21)	36(0.31)	1688(0.25)	346(0.22)	41(0.3)	8(0.26)
Health professional	2929(21.61)	1029(33.59)	236(22.29)	163(20.17)	21(18.26)	1161(17.19)	290(18.24)	21(15.56)	8(25.81)
Pharmacist	778(5.74)	116(3.79)	61(5.76)	27(0.03)	3(0.03)	499(0.07)	61(0.04)	7(0.05)	4(0.13)
Lawyer	331(2.44)	3(0.1)	34(3.21)	30(0.04)	14(0.12)	200(0.03)	39(0.02)	10(0.07)	1(0.03)
Missing	186(1.37)	23(0.75)	19(1.79)	8(0.01)	0(0)	109(0.02)	25(0.02)	1(0.01)	1(0.03)
**History of cardiovascular disease**	2783(20.53)	585	296	170	31	1324	316	46	15
**Outcomes**									
Death	874	37	84	110	9	457	155	13	9
Disability	187	32	14	12	2	108	15	4	0
Hospitalization	4380	513	369	360	51	2428	610	43	6
Life Threatening	513	28	24	18	4	364	68	4	3
Other serious events	5197	887	445	252	42	2872	626	61	12
Required Intervention	48	1	2	1	0	44	0	0	0
Congenital Anomaly	2	1	0	0	0	1	0	0	0
Missing	2355	1564	121	55	7	481	116	10	1

### 3.2 Disproportionality analysis

**Fig 3:**
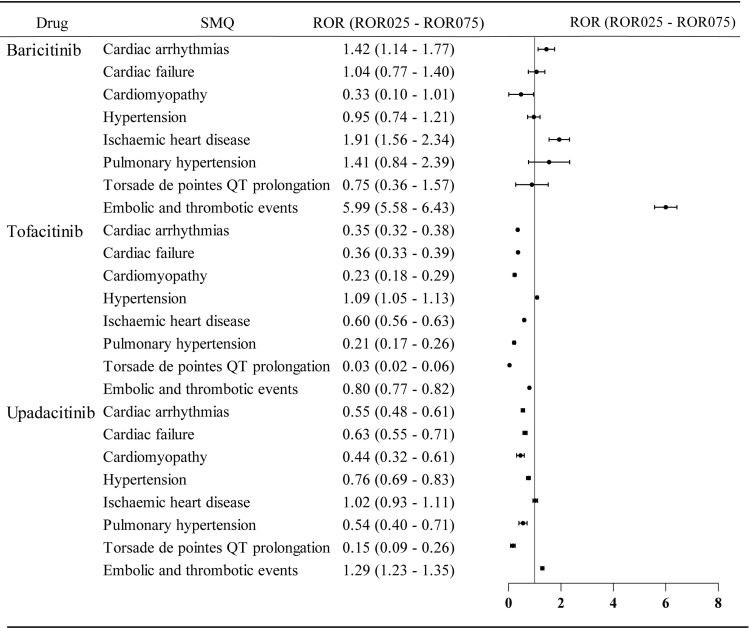
Disproportionality analysis of JAKinibs-related cardiovascular AEs compared to the full database(ROR: reporting odds ratio, ROR025: The lower limit of the 95% CI for ROR, ROR075: The upper limit of the 95%CI for ROR).

A signal was defined as an ROR_025_ value greater than 1 with at least three reports of the specific AE. This criterion indicates a statistically significant association between the drug and the AE. Compared to the entire FAERS database, baricitinib exhibited strong signals for embolic and thrombotic events (ROR_025_ = 5.58), ischemic heart disease (ROR_025_ = 1.56), and cardiac arrhythmias (ROR_025_ = 1.14). Tofacitinib was associated with hypertension (ROR_025_ = 1.05), and upadacitinib was linked to embolic and thrombotic events (ROR_025_ = 1.23) (Fig 3).

**Fig 4:**
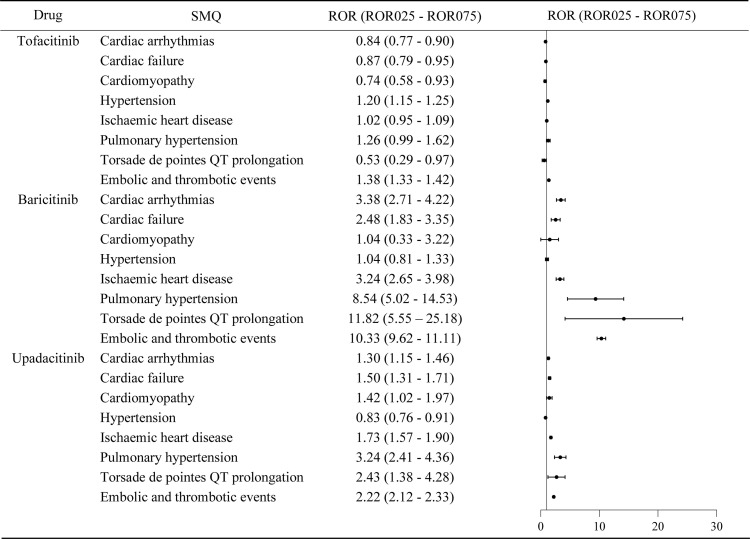
Disproportionality analysis of JAKinibs-related cardiovascular AEs compared to TNF-α inhibitors(ROR: reporting odds ratio, ROR025: The lower limit of the 95% CI for ROR, ROR075: The upper limit of the 95%CI for ROR).

Tofacitinib demonstrated signals for embolic/thrombotic events (ROR_025_ = 1.33) and hypertension (ROR_025_ = 1.15). Baricitinib showed stronger associations with embolic and thrombotic events (ROR_025 _= 9.62), torsade de pointes/QT prolongation (ROR_025 _= 5.55), pulmonary hypertension (ROR_025 _= 5.02), ischemic heart disease(ROR_025 _= 2.65), cardiac arrhythmias (ROR_025 _= 2.71), and cardiac failure (ROR_025 _= 1.83). Upadacitinib was associated with pulmonary hypertension (ROR_025 _= 2.41), embolic and thrombotic events (ROR_025 _= 2.12), ischemic heart disease (ROR_025 _= 1.57), torsade de pointes/QT prolongation (ROR_025 _= 1.38), heart failure (ROR_025 _= 1.31), cardiac arrhythmias (ROR_025 _= 1.15) and cardiomyopathy (ROR_025 _= 1.02) (Fig 4).

We further examined PT signals and found that 40 PTs had signals for baricitinib(S2 Table), 21 PTs had signals for upadacitinib(S3 Table), and 9 PTs had signals for tofacitinib (S4 Table).

### 3.3 Time to onset of TEs

We analyzed 2,175 reports of cardiovascular AEs associated with JAKinibs. The Kruskal-Wallis test results indicated significant differences in onset times across the various SMQs (*p* < 0.001). Median onset times were 179 days (IQR 9.75–359.25) for Torsade de pointes/QT prolongation, 176 days (IQR 20.5–539.5) for Cardiac arrhythmias, 83 days (IQR 15.25–233) for Pulmonary hypertension, 158.5 days (IQR 42–407) for Cardiac failure, 157 days (IQR 39–404) for Cardiomyopathy, 113 days (IQR 14–446) for embolic and thrombotic events, 99 days (IQR 25.25–315.25) for Hypertension, and 278 days (IQR 71.25–631) for Ischemic heart disease.

## 4. Discussion

This study is a pharmacovigilance analysis of cardiovascular AEs associated with JAKinibs, using real-world data from the FAERS database. We found a yearly increase in cardiovascular AE reports linked to JAKinibs. In total, 13,556 cardiovascular AE reports related to JAKinibs were identified in the FAERS database, accounting for 3.1% of all AEs for these drugs.

In the broader context of the tofacitinib RA clinical trial program (P123LTE), which includes 21 phase 1-3b/4 studies and 2 long-term extension studies, no excess risk of MACE was observed [1,7,13]. Observational data and real-world evidence indicated that JAKinibs pose a similar MACE risk in the general RA population when compared to treatment with biosynthetic disease-modifying antirheumatic drugs (bDMARDs) [14–16].The STAR-RA observational study found no evidence that tofacitinib increases the risk of cardiovascular outcomes in real-world RA patients [17]. A systematic review on JAKinibs for atopic dermatitis also found no significant increase in MACE or venous thromboembolism (VTE) risk compared to placebo [18]. However, the ORAL Surveillance study, focusing on RA patients unresponsive to methotrexate, aged over 50, and with at least one cardiovascular risk factor, reported higher MACE incidence with tofacitinib (3.4%) than with TNF-α inhibitors (2.5%) [7].

Dougados et al. noted that P123LTE had fewer patients with a history of atherosclerotic cardiovascular disease (4%) than ORAL Surveillance (15% [13]. Yang et al. cautioned against generalizing ORAL Surveillance results due to its focus on older, higher-risk individuals, affecting external validity [19]. Our study included a high proportion of patients over 50 (71.92%), over 65 (39.19%), and with cardiovascular history (20.53%), aligning with ORAL Surveillance demographics.

Most registration data do not show increased cardiovascular AE risk with baricitinib or upadacitinib. However, baricitinib and upadacitinib share a similar mechanism of action with tofacitinib, and the FDA has expressed concerns that they may pose comparable risks as observed in the safety trials for tofacitinib. In September 2021, the FDA revised the warning for tofacitinib to indicate an increased risk of cardiovascular disease and cancer. It then broadened the black box warning to include other JAK inhibitors, such as baricitinib and upadacitinib, restricting their use in patients who do not respond to TNF-αinhibitors [8]. Our analysis revealed tofacitinib exhibited two positive signals for embolic/thrombotic events and hypertension compared to TNF-α inhibitors. Baricitinib had six positive signals, including torsade de pointes/QT prolongation, embolic and thrombotic events, pulmonary hypertension, ischemic heart disease, arrhythmias, and heart failure. Upadacitinib showed seven positive signals, encompassing pulmonary hypertension, torsade de pointes/QT prolongation, embolic and thrombotic events, ischemic heart disease, heart failure, cardiomyopathy, and arrhythmias. These findings highlight the need for cardiovascular monitoring for patients with pre-existing cardiovascular conditions or other risk factors. The impact of JAKinibs on MACE is complex, involving inflammatory pathways, lipid metabolism, and intricate cytokine signaling. Chronic inflammation increases MACE risk, with the JAK-STAT signaling pathway playing a pivotal role in mediating this inflammation [20–23]. While STAT signaling is generally associated with negative cardiac effects, STAT3 specifically exhibits cardioprotective properties [24–26]. JAK2/STAT3 activation protects against ischemia/reperfusion injury and coronary endothelial cell apoptosis, indicating that JAKinibs could potentially counteract these protective mechanisms [27]. Studies have revealed that treatment with baricitinib resulted in significant increases in both low-density lipoprotein (LDL) and high-density lipoprotein (HDL) levels [28–30]. Research on ulcerative colitis patients treated with tofacitinib also showed notable increases in lipid levels, but without significant alterations in lipid ratios or composite cardiac risk post-treatment [31]. Kotyla et al. suggested that the lipid increases associated with JAKinibs may not increase atherosclerosis risk, potentially due to lipid redistribution rather than synthesis [32]. The impact of IL-6 modulation through JAK inhibition on MACE risk remains incompletely understood, despite IL-6’s correlation with heightened MACE risk and mortality [33]. The specific mechanisms behind JAKinibs’ cardiovascular risk are still under investigation, requiring further research..

This study has several limitations. Firstly, it relied on FAERS AE reports, which may have underreporting, incomplete information, indeterminate causality, and and reporting biases. These factors could impact the accuracy and representativeness of the findings. Secondly, the analysis used spontaneous reporting data, potentially subject to channeling bias, affecting AE reporting accuracy. Thirdly, the study did not account for potential confounding factors not captured in FAERS data, such as lifestyle habits (e.g., smoking and alcohol use), which could contribute to the occurrence of cardiovascular AEs. The absence of this information limits the ability to assess the relationship between these factors and cardiovascular events. Future JAKinib research should focus on large-scale, long-term studies to understand their cardiovascular effects, investigate underlying mechanisms, identify high-risk patients, and compare the cardiovascular safety of different JAKinibs.

## 5. Conclusion

Our analysis of FAERS data reveals a potential association between JAKinibs and increased cardiovascular AE risks, especially in patients with pre-existing cardiovascular conditions or older age. These findings underscore the importance of vigilant cardiovascular monitoring in clinical practice. Physicians are urged to conduct a thorough cardiovascular risk assessment, particularly in older patients or those with cardiovascular risk factors, carefully weighing the potential benefits of treatment against the possible risks.

## Supporting information

S1 Data Two-by-two contingency tables used for calculating ROR. (ZIP)
