# Supplementary material for: Cardiovascular safety of Janus kinase inhibitors: A pharmacovigilance study from 2012–2023
Source: PLoS One. 2025 May 12;20(5):e0322849. doi: 10.1371/journal.pone.0322849 (PMC12068705; doi:10.1371/journal.pone.0322849)
Supplement: S1 Data — (ZIP) [file pone.0322849.s001.zip › Supporting information/S2 Table.docx]

**S2 Table. Signal values of reports associated with baricitinib at the PT level**

| **SMQ** | **PT** | **N** | **ROR** | **ROR_025_** | **ROR_075_** |
| --- | --- | --- | --- | --- | --- |
| Embolic and thrombotic events | Pulmonary Embolism | 236 | 17.342 | 15.237 | 19.738 |
|  | Deep Vein Thrombosis | 168 | 17.48 | 15.006 | 20.372 |
|  | Cerebrovascular Accident | 49 | 2.03 | 1.53 | 2.683 |
|  | Thrombosis | 34 | 2.69 | 1.923 | 3.772 |
|  | Embolism | 21 | 17.11 | 11.14 | 26.276 |
|  | Cerebral Infarction | 20 | 5.35 | 3.449 | 8.3 |
|  | Transient Ischaemic Attack | 19 | 4.08 | 2.604 | 6.408 |
|  | Embolism Venous | 11 | 21.21 | 11.726 | 38.378 |
|  | Thrombophlebitis | 10 | 19.52 | 10.486 | 36.353 |
|  | Retinal Vein Occlusion | 9 | 23.78 | 12.344 | 45.8 |
|  | Pulmonary Infarction | 9 | 27.92 | 14.49 | 53.796 |
|  | Ischaemic Stroke | 8 | 2.67 | 1.333 | 5.336 |
|  | Thrombophlebitis Superficial | 8 | 14.67 | 7.326 | 29.382 |
|  | Peripheral Artery Occlusion | 8 | 30.05 | 14.984 | 60.257 |
|  | Venous Thrombosis | 7 | 11.72 | 5.579 | 24.615 |
|  | Peripheral Artery Thrombosis | 7 | 25.42 | 12.087 | 53.459 |
|  | Hemiparesis | 6 | 2.29 | 1.03 | 5.108 |
|  | Pulmonary Artery Thrombosis | 5 | 45 | 18.636 | 108.669 |
|  | Disseminated Intravascular Coagulation | 5 | 2.76 | 1.15 | 6.646 |
|  | Portal Vein Thrombosis | 4 | 8.43 | 3.16 | 22.486 |
|  | Venous Thrombosis Limb | 4 | 11.91 | 4.463 | 31.788 |
|  | Cardiac Ventricular Thrombosis | 4 | 24.67 | 9.231 | 65.949 |
|  | Cerebral Artery Thrombosis | 4 | 52.37 | 19.528 | 140.451 |
|  | Thrombotic Cerebral Infarction | 3 | 44.43 | 14.24 | 138.653 |
|  | Cerebellar Infarction | 3 | 12.49 | 4.02 | 38.792 |
|  | Splenic Infarction | 3 | 11.53 | 3.712 | 35.811 |
|  | Embolic Stroke | 3 | 4.85 | 1.564 | 15.064 |
|  | Arterial Thrombosis | 3 | 10.72 | 3.451 | 33.286 |
|  | Peripheral Arterial Occlusive Disease | 3 | 4.37 | 1.409 | 13.57 |
|  | Renal Infarct | 3 | 13.53 | 4.354 | 42.031 |
| Cardiac failure | Cardiogenic Shock | 5 | 2.5 | 1.04 | 6.007 |
|  | Cardiac Failure Acute | 4 | 3.92 | 1.471 | 10.457 |
| Cardiac arrhythmias | Pulseless Electrical Activity | 12 | 16.44 | 9.325 | 29 |
|  | Atrioventricular Block Complete | 4 | 4.34 | 1.627 | 11.567 |
|  | Bundle Branch Block Right | 3 | 4.92 | 1.586 | 15.277 |
|  | Atrial Fibrillation | 30 | 1.99 | 1.391 | 2.849 |
| Pulmonary hypertension | Right Ventricular Failure | 5 | 4.14 | 1.724 | 9.965 |
| Ischaemic heart disease | Myocardial Infarction | 40 | 1.71 | 1.255 | 2.336 |
|  | Acute Myocardial Infarction | 28 | 6.38 | 4.403 | 9.252 |
|  | Acute Coronary Syndrome | 5 | 3.92 | 1.632 | 9.431 |
