# Supplementary material for: Cardiovascular safety of Janus kinase inhibitors: A pharmacovigilance study from 2012–2023
Source: PLoS One. 2025 May 12;20(5):e0322849. doi: 10.1371/journal.pone.0322849 (PMC12068705; doi:10.1371/journal.pone.0322849)
Supplement: S1 Data — (ZIP) [file pone.0322849.s001.zip › Supporting information/S1 Table.docx]

**S1 Table. Two-by-two contingency tables used for calculating ROR**

|  | JAKinibs | All Other Drugs | Total | |
| --- | --- | --- | --- | --- |
| Adverse event | *a* | *b* | | *a+b* |
| All other adverse events | *c* | *d* | | *c+d* |
| Total | *a+c* | *b+d* | | *a+b+c+d* |

a = The number of reports of JAKinibs with the adverse event of interest. b = The number of reports of all other drugs with the adverse event of interest. c = The number of reports of JAKinibs with all other adverse events. d = The number of reports of all other drugs with all other adverse events.
