# Supplementary material for: Cardiovascular safety of Janus kinase inhibitors: A pharmacovigilance study from 2012–2023
Source: PLoS One. 2025 May 12;20(5):e0322849. doi: 10.1371/journal.pone.0322849 (PMC12068705; doi:10.1371/journal.pone.0322849)
Supplement: S1 Data — (ZIP) [file pone.0322849.s001.zip › Supporting information/S4 Table.docx]

**S4 Table. Signal values of reports associated with tofacitinib at the PT level**

| **SMQ** | **PT** | **N** | **ROR** | **ROR_025_** | **ROR_075_** |
| --- | --- | --- | --- | --- | --- |
| Embolic and thrombotic events | Retinal Vein Thrombosis | 9 | 3.219 | 1.66 | 6.244 |
|  | Post Procedural Pulmonary Embolism | 4 | 2.954 | 1.095 | 7.97 |
|  | Thrombosis | 765 | 1.733 | 1.613 | 1.861 |
|  | Pulmonary Embolism | 549 | 1.119 | 1.029 | 1.217 |
|  | Pulmonary Thrombosis | 137 | 2.193 | 1.852 | 2.598 |
| Hypertension | Diastolic Hypertension | 3 | 4.519 | 1.425 | 14.324 |
|  | Hypertension | 1308 | 1.147 | 1.086 | 1.211 |
|  | Blood Pressure Increased | 1112 | 1.309 | 1.234 | 1.389 |
| Ischaemic heart disease | Coronary Artery Thrombosis | 14 | 1.742 | 1.028 | 2.953 |
