# Supplementary material for: Cardiovascular safety of Janus kinase inhibitors: A pharmacovigilance study from 2012–2023
Source: PLoS One. 2025 May 12;20(5):e0322849. doi: 10.1371/journal.pone.0322849 (PMC12068705; doi:10.1371/journal.pone.0322849)
Supplement: S1 Data — (ZIP) [file pone.0322849.s001.zip › Supporting information/S3 Table.docx]

**S3 Table. Signal values of reports associated with upadacitinib at the PT level**

| **SMQ** | **PT** | **N** | **ROR** | **ROR_025_** | **ROR_075_** |
| --- | --- | --- | --- | --- | --- |
| Embolic and thrombotic events | Left Atrial Appendage Closure Implant | 3 | 84.148 | 24.518 | 288.802 |
|  | Arterial Therapeutic Procedure | 4 | 13.009 | 4.814 | 35.154 |
|  | Venous Operation | 3 | 12.021 | 3.819 | 37.839 |
|  | Hepatic Vascular Thrombosis | 3 | 10.685 | 3.4 | 33.579 |
|  | Postoperative Thrombosis | 7 | 8.584 | 4.063 | 18.134 |
|  | Pulmonary Thrombosis | 96 | 5.996 | 4.902 | 7.334 |
|  | Vascular Operation | 3 | 5.407 | 1.732 | 16.88 |
|  | Venous Thrombosis Limb | 15 | 4.98 | 2.994 | 8.284 |
|  | Embolism Venous | 22 | 4.714 | 3.097 | 7.175 |
|  | Retinal Vein Occlusion | 12 | 3.511 | 1.989 | 6.196 |
|  | Peripheral Artery Occlusion | 8 | 3.321 | 1.657 | 6.659 |
|  | Venous Occlusion | 7 | 2.647 | 1.259 | 5.564 |
|  | Carotid Artery Occlusion | 12 | 2.56 | 1.451 | 4.515 |
|  | Thrombosis | 289 | 2.545 | 2.266 | 2.857 |
|  | Arterial Occlusive Disease | 23 | 2.29 | 1.52 | 3.45 |
|  | Cerebral Thrombosis | 8 | 2.122 | 1.059 | 4.25 |
|  | Transient Ischaemic Attack | 83 | 1.979 | 1.595 | 2.455 |
|  | Cerebrovascular Accident | 299 | 1.369 | 1.222 | 1.533 |
| Ischaemic heart disease | Coronary Arterial Stent Insertion | 24 | 3.059 | 2.047 | 4.57 |
|  | Coronary Artery Occlusion | 41 | 2.537 | 1.866 | 3.449 |
|  | Myocardial Infarction | 254 | 1.204 | 1.064 | 1.362 |
